# Supplementary material for: Low n-6/n-3 Gestation and Lactation Diets Influence Early Performance, Muscle and Adipose Polyunsaturated Fatty Acid Content and Deposition, and Relative Abundance of Proteins in Suckling Piglets
Source: Molecules. 2022 May 4;27(9):2925. doi: 10.3390/molecules27092925 (PMC9103047; doi:10.3390/molecules27092925)
Supplement: Supplementary file 1 [file molecules-27-02925-s001.zip › molecules-1667351-supplementary.pdf]

Supplementary materials

Table S1. The abundance ratio of *longissimus dorsi* muscle proteins derived from suckling piglets.

UNIPROT Accession number	Description	Abundance Ratio: (soya) / (lino)	Abundance Ratio Adj. P-Value: (soya) / (lino)
A7VK00	Interferon-induced GTP-binding protein Mx2 [OS=Sus scrofa]	100.000	6.50E-16
P51524	Prophenin and tritrpticin precursor [OS=Sus scrofa]	100.000	6.50E-16
Q6RI85	Phosphoglycerate kinase 2 [OS=Sus scrofa]	5.117	1.73E-08
Q8SPS7	Haptoglobin [OS=Sus scrofa]	2.445	1.24E-02
P02189	Myoglobin [OS=Sus scrofa]	2.097	6.26E-02
Q29550	Liver carboxylesterase [OS=Sus scrofa]	2.023	9.28E-02
P32196	Protegrin-3 [OS=Sus scrofa]	2.001	9.55E-02
P80310	Protein S100-A12 [OS=Sus scrofa]	1.986	1.03E-01
P56472	Isocitrate dehydrogenase [NAD] subunit beta, mitochondrial [OS=Sus scrofa]	1.724	3.42E-01
Q9MYT8	ATP synthase subunit e, mitochondrial [OS=Sus scrofa]	1.715	3.50E-01
Q5S3G4	Cytochrome c oxidase subunit 5B, mitochondrial [OS=Sus scrofa]	1.667	4.26E-01
Q8MJ14	Glutathione peroxidase 1 [OS=Sus scrofa]	1.663	4.30E-01
P53590	Succinate--CoA ligase [GDP-forming] subunit beta, mitochondrial [OS=Sus scrofa]	1.654	4.47E-01
Q29042	Ficolin-1 [OS=Sus scrofa]	1.646	4.55E-01
P80229	Leukocyte elastase inhibitor [OS=Sus scrofa]	1.623	4.81E-01
Q6UAQ8	Electron transfer flavoprotein subunit beta [OS=Sus scrofa]	1.619	4.83E-01
P41566	Isocitrate dehydrogenase [NAD] subunit gamma, mitochondrial [OS=Sus scrofa]	1.619	4.83E-01
P79293	Myosin-7 [OS=Sus scrofa]	1.581	5.54E-01
P79263	Inter-alpha-trypsin inhibitor heavy chain H4 [OS=Sus scrofa]	1.573	5.73E-01
Q2EN81	ATP synthase subunit O, mitochondrial [OS=Sus scrofa]	1.570	5.79E-01
P81271	Myosin-11 [OS=Sus scrofa]	1.566	5.87E-01
Q29195	60S ribosomal protein L10 [OS=Sus scrofa]	1.564	5.87E-01
P41367	Medium-chain specific acyl-CoA dehydrogenase, mitochondrial [OS=Sus scrofa]	1.564	5.87E-01

P09623	Dihydrolipoyl dehydrogenase, mitochondrial [OS=Sus scrofa]	1.558	5.87E-01
P79273	Short-chain specific acyl-CoA dehydrogenase, mitochondrial [OS=Sus scrofa]	1.549	6.03E-01
P00348	Hydroxyacyl-coenzyme A dehydrogenase, mitochondrial [OS=Sus scrofa]	1.529	6.38E-01
P02540	Desmin [OS=Sus scrofa]	1.514	6.69E-01
P13618	ATP synthase-coupling factor 6, mitochondrial [OS=Sus scrofa]	1.512	6.73E-01
P00346	Malate dehydrogenase, mitochondrial [OS=Sus scrofa]	1.496	6.83E-01
P02067	Hemoglobin subunit beta [OS=Sus scrofa]	1.491	6.83E-01
P79274	Long-chain specific acyl-CoA dehydrogenase, mitochondrial [OS=Sus scrofa]	1.489	6.87E-01
Q29554	Trifunctional enzyme subunit alpha, mitochondrial [OS=Sus scrofa]	1.486	6.87E-01
Q35914	ATP synthase protein 8 [OS=Sus scrofa]	1.485	6.87E-01
O19069	Succinate--CoA ligase [ADP/GDP-forming] subunit alpha, mitochondrial [OS=Sus scrofa]	1.485	6.87E-01
P80021	ATP synthase subunit alpha, mitochondrial [OS=Sus scrofa]	1.482	6.93E-01
P14610	Acetyl-CoA acetyltransferase [OS=Sus scrofa]	1.476	7.04E-01
Q95339	ATP synthase subunit f, mitochondrial [OS=Sus scrofa]	1.471	7.04E-01
Q9TV63	Myosin-2 [OS=Sus scrofa]	1.466	7.10E-01
Q8WNV7	Dehydrogenase/reductase SDR family member 4 [OS=Sus scrofa]	1.454	7.33E-01
Q29236	T-complex protein 1 subunit zeta [OS=Sus scrofa]	1.436	7.71E-01
P81140	Glutaryl-CoA dehydrogenase, mitochondrial [OS=Sus scrofa]	1.432	7.74E-01
P55931	Electron transfer flavoprotein-ubiquinone oxidoreductase, mitochondrial [OS=Sus scrofa]	1.428	7.79E-01
D2SW95	Coatomer subunit beta [OS=Sus scrofa]	1.427	7.88E-01
P29804	Pyruvate dehydrogenase E1 component subunit alpha, somatic form, mitochondrial [OS=Sus scrofa]	1.405	8.12E-01
A9YUB1	Cysteine and histidine-rich domain-containing protein 1 [OS=Sus scrofa]	1.388	8.54E-01
P0DTA4	Propionyl-CoA carboxylase alpha chain, mitochondrial [OS=Sus scrofa]	1.388	8.54E-01
Q9GLP0	Integrin beta-1 [OS=Sus scrofa]	1.385	8.57E-01
Q8MJ30	Dihydropteridine reductase [OS=Sus scrofa]	1.383	8.63E-01
Q6QAP7	40S ribosomal protein S17 [OS=Sus scrofa]	1.379	8.70E-01

P56471	Isocitrate dehydrogenase [NAD] subunit alpha, mitochondrial [OS=Sus scrofa]	1.378	8.71E-01
P01965	Hemoglobin subunit alpha [OS=Sus scrofa]	1.370	8.77E-01
Q007T0	Succinate dehydrogenase [ubiquinone] iron-sulfur subunit, mitochondrial [OS=Sus scrofa]	1.369	8.77E-01
P51781	Glutathione S-transferase alpha M14 [OS=Sus scrofa]	1.368	8.77E-01
Q06AA3	Regucalcin [OS=Sus scrofa]	1.367	8.77E-01
P00506	Aspartate aminotransferase, mitochondrial [OS=Sus scrofa]	1.364	8.77E-01
Q0MVN8	Quinone oxidoreductase [OS=Sus scrofa]	1.364	8.77E-01
P14632	Lactotransferrin [OS=Sus scrofa]	1.362	8.79E-01
Q4PLW0	Perilipin-2 [OS=Sus scrofa]	1.361	8.79E-01
Q64L94	Proteasome activator complex subunit 1 [OS=Sus scrofa]	1.349	8.85E-01
Q75ZZ6	Troponin T, slow skeletal muscle [OS=Sus scrofa]	1.344	8.85E-01
P79403	Neutral alpha-glucosidase AB [OS=Sus scrofa]	1.343	8.85E-01
P10173	Fumarate hydratase, mitochondrial [OS=Sus scrofa]	1.339	8.85E-01
Q0QF01	Succinate dehydrogenase [ubiquinone] flavoprotein subunit, mitochondrial [OS=Sus scrofa]	1.338	8.85E-01
Q863Z0	Proteasome activator complex subunit 2 [OS=Sus scrofa]	1.334	8.85E-01
P50667	Cytochrome c oxidase subunit 2 [OS=Sus scrofa]	1.333	8.85E-01
Q28948	5'-AMP-activated protein kinase catalytic subunit alpha-2 [OS=Sus scrofa]	1.331	8.85E-01
Q9XT00	Estradiol 17-beta-dehydrogenase 8 [OS=Sus scrofa]	1.330	8.85E-01
P16276	Aconitate hydratase, mitochondrial [OS=Sus scrofa]	1.328	8.85E-01
Q29551	Succinyl-CoA:3-ketoacid coenzyme A transferase 1, mitochondrial [OS=Sus scrofa]	1.327	8.85E-01
Q7M2W6	Alpha-crystallin B chain [OS=Sus scrofa]	1.319	8.92E-01
Q2L969	Metaxin-2 [OS=Sus scrofa]	1.317	8.92E-01
P53027	60S ribosomal protein L10a [OS=Sus scrofa]	1.313	8.92E-01
P05207	cAMP-dependent protein kinase type II-alpha regulatory subunit [OS=Sus scrofa]	1.312	8.92E-01
P33198	Isocitrate dehydrogenase [NADP], mitochondrial [OS=Sus scrofa]	1.312	8.92E-01
P04366	Protein AMBP [OS=Sus scrofa]	1.311	8.92E-01
Q9N0F1	Dihydrolipoyllysine-residue succinyltransferase component of 2-oxoglutarate dehydrogenase complex, mitochondrial [OS=Sus scrofa]	1.310	8.94E-01
P28768	Superoxide dismutase [Mn], mitochondrial [OS=Sus scrofa]	1.303	8.96E-01

O97580	Succinate--CoA ligase [ADP-forming] subunit beta, mitochondrial [OS=Sus scrofa]	1.295	9.14E-01
P00336	L-lactate dehydrogenase B chain [OS=Sus scrofa]	1.279	9.18E-01
P20112	SPARC [OS=Sus scrofa]	1.276	9.18E-01
P79384	Propionyl-CoA carboxylase beta chain, mitochondrial [OS=Sus scrofa]	1.271	9.18E-01
Q6DUB7	Stathmin [OS=Sus scrofa]	1.265	9.18E-01
Q95332	Betaine--homocysteine S-methyltransferase 1 [OS=Sus scrofa]	1.258	9.18E-01
Q29381	Dolichyl-diphosphooligosaccharide--protein glycosyltransferase 48 kDa subunit [OS=Sus scrofa]	1.253	9.19E-01
Q6RVA9	Caveolin-1 [OS=Sus scrofa]	1.246	9.30E-01
P0C5I2	Mannose-1-phosphate guanyltransferase beta [OS=Sus scrofa]	1.246	9.34E-01
P79376	Metallothionein-1C [OS=Sus scrofa]	1.238	9.34E-01
P19205	Acylamino-acid-releasing enzyme [OS=Sus scrofa]	1.236	9.34E-01
O02668	Inter-alpha-trypsin inhibitor heavy chain H2 [OS=Sus scrofa]	1.236	9.34E-01
Q29308	40S ribosomal protein S19 [OS=Sus scrofa]	1.235	9.34E-01
Q06AU7	Ras-related protein Rab-1B [OS=Sus scrofa]	1.232	9.54E-01
Q7YS99	Optineurin [OS=Sus scrofa]	1.228	9.41E-01
Q5GN48	Dystrophin [OS=Sus scrofa]	1.221	9.54E-01
P11708	Malate dehydrogenase, cytoplasmic [OS=Sus scrofa]	1.218	9.58E-01
A5GFS8	Vesicle-associated membrane protein-associated protein B [OS=Sus scrofa]	1.214	9.64E-01
P47788	Thimet oligopeptidase [OS=Sus scrofa]	1.213	9.64E-01
Q29205	60S ribosomal protein L11 [OS=Sus scrofa]	1.212	9.64E-01
P01025	Complement C3 [OS=Sus scrofa]	1.211	9.64E-01
D2WKD8	Sodium/potassium-transporting ATPase subunit alpha-2 [OS=Sus scrofa]	1.210	9.64E-01
P46410	Glutamine synthetase [OS=Sus scrofa]	1.208	9.64E-01
Q19AZ8	Prothrombin [OS=Sus scrofa]	1.208	9.64E-01
Q06AA9	Ubiquitin-conjugating enzyme E2 D2 [OS=Sus scrofa]	1.208	9.64E-01
Q29259	NADH dehydrogenase [ubiquinone] 1 beta subcomplex subunit 6 [OS=Sus scrofa]	1.207	9.64E-01
Q52NJ6	Ras-related protein Rab-14 [OS=Sus scrofa]	1.205	9.64E-01
P02543	Vimentin [OS=Sus scrofa]	1.204	9.64E-01
P07802	cAMP-dependent protein kinase type I-alpha regulatory subunit [OS=Sus scrofa]	1.203	9.64E-01
P10668	Cofilin-1 [OS=Sus scrofa]	1.203	9.64E-01

P42174	Glutamate dehydrogenase 1, mitochondrial [OS=Sus scrofa]	1.203	9.64E-01
Q007T2	Cell division control protein 42 homolog [OS=Sus scrofa]	1.195	9.70E-01
Q28960	Carbonyl reductase [NADPH] 1 [OS=Sus scrofa]	1.190	9.74E-01
A1XQV4	Tropomyosin alpha-3 chain [OS=Sus scrofa]	1.190	9.74E-01
P19619	Annexin A1 [OS=Sus scrofa]	1.188	9.74E-01
P05027	Sodium/potassium-transporting ATPase subunit beta-1 [OS=Sus scrofa]	1.188	9.74E-01
P48819	Vitronectin [OS=Sus scrofa]	1.188	9.74E-01
P79324	60S ribosomal protein L15 [OS=Sus scrofa]	1.185	9.75E-01
P62197	26S proteasome regulatory subunit 8 [OS=Sus scrofa]	1.184	9.75E-01
P80928	Macrophage migration inhibitory factor [OS=Sus scrofa]	1.179	9.76E-01
D0VWV4	Succinate dehydrogenase cytochrome b560 subunit, mitochondrial [OS=Sus scrofa]	1.179	9.76E-01
P28839	Cytosol aminopeptidase [OS=Sus scrofa]	1.175	9.77E-01
P80895	Protein-L-isoaspartate(D-aspartate) O-methyltransferase [OS=Sus scrofa]	1.175	9.77E-01
P06867	Plasminogen [OS=Sus scrofa]	1.174	9.77E-01
P00889	Citrate synthase, mitochondrial [OS=Sus scrofa]	1.172	9.81E-01
Q5S1U1	Heat shock protein beta-1 [OS=Sus scrofa]	1.170	9.81E-01
P46405	40S ribosomal protein S12 [OS=Sus scrofa]	1.169	9.81E-01
P26042	Moesin [OS=Sus scrofa]	1.167	9.81E-01
O62839	Catalase [OS=Sus scrofa]	1.164	9.81E-01
Q99028	Catechol O-methyltransferase [OS=Sus scrofa]	1.159	9.81E-01
Q6QRN9	ADP/ATP translocase 3 [OS=Sus scrofa]	1.156	9.90E-01
Q95283	Cytochrome c oxidase subunit 4 isoform 1, mitochondrial [OS=Sus scrofa]	1.153	9.90E-01
Q9TV62	Myosin-4 [OS=Sus scrofa]	1.151	9.90E-01
P63317	Troponin C, slow skeletal and cardiac muscles [OS=Sus scrofa]	1.150	9.90E-01
A1XQT2	Cytochrome c oxidase subunit 6C [OS=Sus scrofa]	1.145	1.00E+00
Q29545	Inhibitor of carbonic anhydrase [OS=Sus scrofa]	1.144	1.00E+00
Q0MUU2	Small muscular protein [OS=Sus scrofa]	1.143	1.00E+00
Q767L7	Tubulin beta chain [OS=Sus scrofa]	1.141	1.00E+00
P54612	Serine/threonine-protein phosphatase 2A 65 kDa regulatory subunit A alpha isoform [OS=Sus scrofa]	1.139	1.00E+00
P00503	Aspartate aminotransferase, cytoplasmic [OS=Sus scrofa]	1.138	1.00E+00

Q9MYY8	Thioredoxin reductase 1, cytoplasmic [OS=Sus scrofa]	1.137	1.00E+00
Q5PXT2	LIM and cysteine-rich domains protein 1 [OS=Sus scrofa]	1.133	1.00E+00
Q6SEG5	Ubiquitin carboxyl-terminal hydrolase isozyme L1 [OS=Sus scrofa]	1.133	1.00E+00
Q29307	ATPase inhibitor, mitochondrial [OS=Sus scrofa]	1.132	1.00E+00
Q29549	Clusterin [OS=Sus scrofa]	1.131	1.00E+00
Q9XSD9	Decorin [OS=Sus scrofa]	1.131	1.00E+00
Q764M5	Signal transducer and activator of transcription 1 [OS=Sus scrofa]	1.129	1.00E+00
Q2XVP4	Tubulin alpha-1B chain [OS=Sus scrofa]	1.128	1.00E+00
Q8HY46	Carnitine O-palmitoyltransferase 1, muscle isoform [OS=Sus scrofa]	1.127	1.00E+00
P67872	Casein kinase II subunit beta [OS=Sus scrofa]	1.125	1.00E+00
Q9GKX6	Galactose mutarotase [OS=Sus scrofa]	1.125	1.00E+00
P17741	High mobility group protein B2 [OS=Sus scrofa]	1.125	1.00E+00
P12682	High mobility group protein B1 [OS=Sus scrofa]	1.121	1.00E+00
Q5D144	Transcription factor A, mitochondrial [OS=Sus scrofa]	1.121	1.00E+00
P79303	UTP--glucose-1-phosphate uridylyltransferase [OS=Sus scrofa]	1.120	1.00E+00
P50447	Alpha-1-antitrypsin [OS=Sus scrofa]	1.119	1.00E+00
Q29243	Dystroglycan [OS=Sus scrofa]	1.119	1.00E+00
Q9GJT2	S-formylglutathione hydrolase [OS=Sus scrofa]	1.118	1.00E+00
P36887	cAMP-dependent protein kinase catalytic subunit alpha [OS=Sus scrofa]	1.117	1.00E+00
O46560	Pyridoxal kinase [OS=Sus scrofa]	1.117	1.00E+00
Q29024	Spliceosome RNA helicase DDX39B [OS=Sus scrofa]	1.117	1.00E+00
P82460	Thioredoxin [OS=Sus scrofa]	1.117	1.00E+00
P45845	Protein-lysine 6-oxidase [OS=Sus scrofa]	1.114	1.00E+00
Q4PS85	Myozenin-1 [OS=Sus scrofa]	1.112	1.00E+00
P23687	Prolyl endopeptidase [OS=Sus scrofa]	1.106	1.00E+00
P81693	Low molecular weight phosphotyrosine protein phosphatase [OS=Sus scrofa]	1.103	1.00E+00
P18650	Apolipoprotein E [OS=Sus scrofa]	1.101	1.00E+00
Q9GMB0	Dolichyl-diphosphooligosaccharide--protein glycosyltransferase subunit 1 [OS=Sus scrofa]	1.099	1.00E+00
Q29387	Elongation factor 1-gamma [OS=Sus scrofa]	1.099	1.00E+00
P03974	Transitional endoplasmic reticulum ATPase [OS=Sus scrofa]	1.099	1.00E+00
P00819	Acylphosphatase-2 [OS=Sus scrofa]	1.098	1.00E+00

P63221	40S ribosomal protein S21 [OS=Sus scrofa]	1.097	1.00E+00
Q4GWZ2	40S ribosomal protein SA [OS=Sus scrofa]	1.094	1.00E+00
O02705	Heat shock protein HSP 90-alpha [OS=Sus scrofa]	1.094	1.00E+00
P83686	NADH-cytochrome b5 reductase 3 [OS=Sus scrofa]	1.093	1.00E+00
Q52NJ3	GTP-binding protein SAR1a [OS=Sus scrofa]	1.092	1.00E+00
Q9TTB4	Fibromodulin [OS=Sus scrofa]	1.091	1.00E+00
P10775	Ribonuclease inhibitor [OS=Sus scrofa]	1.089	1.00E+00
Q29561	UMP-CMP kinase [OS=Sus scrofa]	1.086	1.00E+00
Q95276	60S ribosomal protein L5 [OS=Sus scrofa]	1.084	1.00E+00
Q29099	Polypyrimidine tract-binding protein 1 [OS=Sus scrofa]	1.083	1.00E+00
Q29577	Creatine kinase U-type, mitochondrial [OS=Sus scrofa]	1.081	1.00E+00
P67776	Serine/threonine-protein phosphatase 2A catalytic subunit alpha isoform [OS=Sus scrofa]	1.080	1.00E+00
Q29092	Endoplasmin [OS=Sus scrofa]	1.079	1.00E+00
Q49I35	Galectin-1 [OS=Sus scrofa]	1.079	1.00E+00
A1E295	Cathepsin B [OS=Sus scrofa]	1.073	1.00E+00
Q8SPR7	1-phosphatidylinositol 4,5-bisphosphate phosphodiesterase delta-4 [OS=Sus scrofa]	1.072	1.00E+00
Q6Q7J2	Rab GDP dissociation inhibitor beta [OS=Sus scrofa]	1.072	1.00E+00
P68137	Actin, alpha skeletal muscle [OS=Sus scrofa]	1.069	1.00E+00
F1RRT2	Myosin light chain 4 [OS=Sus scrofa]	1.069	1.00E+00
P16960	Ryanodine receptor 1 [OS=Sus scrofa]	1.068	1.00E+00
Q9TV69	Trans-1,2-dihydrobenzene-1,2-diol dehydrogenase [OS=Sus scrofa]	1.067	1.00E+00
Q29238	Chloride intracellular channel protein 1 [OS=Sus scrofa]	1.066	1.00E+00
Q08092	Calponin-1 [OS=Sus scrofa]	1.063	1.00E+00
Q71U53	cAMP-dependent protein kinase inhibitor alpha [OS=Sus scrofa]	1.063	1.00E+00
P04178	Superoxide dismutase [Cu-Zn] [OS=Sus scrofa]	1.063	1.00E+00
P62272	40S ribosomal protein S18 [OS=Sus scrofa]	1.060	1.00E+00
Q710C4	Adenosylhomocysteinase [OS=Sus scrofa]	1.058	1.00E+00
P81405	Saposin-B-Val [OS=Sus scrofa]	1.057	1.00E+00
Q29214	60S acidic ribosomal protein P0 [OS=Sus scrofa]	1.054	1.00E+00
Q6QGC0	PDZ and LIM domain protein 3 [OS=Sus scrofa]	1.054	1.00E+00
P29700	Alpha-2-HS-glycoprotein [OS=Sus scrofa]	1.052	1.00E+00
P05024	Sodium/potassium-transporting ATPase subunit alpha-1 [OS=Sus scrofa]	1.052	1.00E+00
P26234	Vinculin [OS=Sus scrofa]	1.047	1.00E+00

P62936	Peptidyl-prolyl cis-trans isomerase A [OS=Sus scrofa]	1.046	1.00E+00
Q6S4N2	Heat shock 70 kDa protein 1B [OS=Sus scrofa]	1.044	1.00E+00
P61292	Serine/threonine-protein phosphatase PP1-beta catalytic subunit [OS=Sus scrofa]	1.043	1.00E+00
Q71LE2	Histone H3.3 [OS=Sus scrofa]	1.041	1.00E+00
P62863	40S ribosomal protein S30 [OS=Sus scrofa]	1.039	1.00E+00
P35750	Calpain-1 catalytic subunit [OS=Sus scrofa]	1.039	1.00E+00
O02772	Fatty acid-binding protein, heart [OS=Sus scrofa]	1.038	1.00E+00
P59083	14 kDa phosphohistidine phosphatase [OS=Sus scrofa]	1.036	1.00E+00
P21753	Thymosin beta-10 [OS=Sus scrofa]	1.035	1.00E+00
A9CQL8	Prostamide/prostaglandin F synthase [OS=Sus scrofa]	1.034	1.00E+00
Q3YLA6	Serine/arginine-rich splicing factor 1 [OS=Sus scrofa]	1.027	1.00E+00
Q95266	Calcium/calmodulin-dependent protein kinase type II subunit delta [OS=Sus scrofa]	1.026	1.00E+00
P28491	Calreticulin [OS=Sus scrofa]	1.025	1.00E+00
Q9MZ15	Voltage-dependent anion-selective channel protein 2 [OS=Sus scrofa]	1.025	1.00E+00
Q0Z8U2	40S ribosomal protein S3 [OS=Sus scrofa]	1.024	1.00E+00
Q29201	40S ribosomal protein S16 [OS=Sus scrofa]	1.023	1.00E+00
P31950	Protein S100-A11 [OS=Sus scrofa]	1.022	1.00E+00
Q5XLD3	Creatine kinase M-type [OS=Sus scrofa]	1.016	1.00E+00
Q95342	60S ribosomal protein L18 [OS=Sus scrofa]	1.015	1.00E+00
Q3ZD69	Prelamin-A/C [OS=Sus scrofa]	1.014	1.00E+00
O46409	Apolipoprotein A-IV [OS=Sus scrofa]	1.013	1.00E+00
Q06A98	Serine/arginine-rich splicing factor 2 [OS=Sus scrofa]	1.011	1.00E+00
P37111	Aminoacylase-1 [OS=Sus scrofa]	1.009	1.00E+00
P04574	Calpain small subunit 1 [OS=Sus scrofa]	1.005	1.00E+00
A7TX81	COP9 signalosome complex subunit 6 [OS=Sus scrofa]	1.005	1.00E+00
P02550	Tubulin alpha-1A chain [OS=Sus scrofa]	1.001	1.00E+00
P80276	Aldo-keto reductase family 1 member B1 [OS=Sus scrofa]	0.999	1.00E+00
P61013	Cardiac phospholamban [OS=Sus scrofa]	0.993	9.90E-01
P62802	Histone H4 [OS=Sus scrofa]	0.992	9.90E-01
Q9TSX9	Peroxiredoxin-6 [OS=Sus scrofa]	0.991	9.90E-01
P19620	Annexin A2 [OS=Sus scrofa]	0.985	9.81E-01
Q29599	2'-5'-oligoadenylate synthase 1 [OS=Sus scrofa]	0.984	9.81E-01

Q29384	Proteasome subunit beta type-4 [OS=Sus scrofa]	0.984	9.81E-01
Q9GKQ6	Biglycan [OS=Sus scrofa]	0.983	9.81E-01
A7Y521	COP9 signalosome complex subunit 4 [OS=Sus scrofa]	0.981	9.81E-01
Q56P28	PRA1 family protein 3 [OS=Sus scrofa]	0.981	9.81E-01
Q29090	Serine/threonine-protein phosphatase 2A 55 kDa regulatory subunit B alpha isoform [OS=Sus scrofa]	0.973	9.77E-01
P49171	40S ribosomal protein S26 [OS=Sus scrofa]	0.970	9.76E-01
P61288	Translationally-controlled tumor protein [OS=Sus scrofa]	0.968	9.76E-01
P63246	Receptor of activated protein C kinase 1 [OS=Sus scrofa]	0.967	9.76E-01
P18648	Apolipoprotein A-I [OS=Sus scrofa]	0.965	9.76E-01
P02554	Tubulin beta chain [OS=Sus scrofa]	0.965	9.75E-01
A0PFK7	F-actin-capping protein subunit beta [OS=Sus scrofa]	0.961	9.74E-01
P09571	Serotransferrin [OS=Sus scrofa]	0.960	9.74E-01
Q8MIK9	Protein phosphatase 1 regulatory subunit 14B [OS=Sus scrofa]	0.959	9.74E-01
Q29228	4-trimethylaminobutyraldehyde dehydrogenase [OS=Sus scrofa]	0.956	9.70E-01
P60982	Destrin [OS=Sus scrofa]	0.955	9.70E-01
Q6QAT1	40S ribosomal protein S28 [OS=Sus scrofa]	0.954	9.68E-01
Q29221	F-actin-capping protein subunit alpha-2 [OS=Sus scrofa]	0.954	9.68E-01
A1XQU9	40S ribosomal protein S20 [OS=Sus scrofa]	0.952	9.68E-01
P52552	Peroxiredoxin-2 [OS=Sus scrofa]	0.949	9.64E-01
P04163	Protein S100-A10 [OS=Sus scrofa]	0.945	9.64E-01
P60662	Myosin light polypeptide 6 [OS=Sus scrofa]	0.944	9.64E-01
Q29380	Voltage-dependent anion-selective channel protein 3 [OS=Sus scrofa]	0.936	9.54E-01
Q9TV61	Myosin-1 [OS=Sus scrofa]	0.935	9.54E-01
P11607	Sarcoplasmic/endoplasmic reticulum calcium ATPase 2 [OS=Sus scrofa]	0.934	9.53E-01
P29269	Myosin regulatory light polypeptide 9 [OS=Sus scrofa]	0.933	9.53E-01
Q2XQV4	Aldehyde dehydrogenase, mitochondrial [OS=Sus scrofa]	0.926	9.37E-01
Q9N1F5	Glutathione S-transferase omega-1 [OS=Sus scrofa]	0.924	9.34E-01
Q462R2	Integrin beta-1-binding protein 2 [OS=Sus scrofa]	0.924	9.34E-01
P86412	Myelin P2 protein [OS=Sus scrofa]	0.922	9.34E-01

O77696	Sarcoplasmic/endoplasmic reticulum calcium ATPase 3 [OS=Sus scrofa]	0.919	9.33E-01
P11493	Serine/threonine-protein phosphatase 2A catalytic subunit beta isoform [OS=Sus scrofa]	0.918	9.18E-01
P26044	Radixin [OS=Sus scrofa]	0.917	9.30E-01
Q19PY3	RNA-splicing ligase RtcB homolog [OS=Sus scrofa]	0.915	9.25E-01
O11780	Transforming growth factor-beta-induced protein ig-h3 [OS=Sus scrofa]	0.911	9.18E-01
P24540	Acylphosphatase-1 [OS=Sus scrofa]	0.909	9.18E-01
P08552	Neurofilament medium polypeptide [OS=Sus scrofa]	0.909	9.18E-01
P67937	Tropomyosin alpha-4 chain [OS=Sus scrofa]	0.909	9.18E-01
P20305	Gelsolin [OS=Sus scrofa]	0.907	9.18E-01
P42639	Tropomyosin alpha-1 chain [OS=Sus scrofa]	0.907	9.18E-01
P61220	Eukaryotic translation initiation factor 1b [OS=Sus scrofa]	0.906	9.18E-01
Q52NJ1	Ras-related protein Rab-11A [OS=Sus scrofa]	0.906	9.18E-01
P02547	Neurofilament light polypeptide [OS=Sus scrofa]	0.905	9.18E-01
P43368	Calpain-3 [OS=Sus scrofa]	0.904	9.18E-01
Q9GL51	Platelet-activating factor acetylhydrolase IB subunit alpha [OS=Sus scrofa]	0.904	9.18E-01
Q8HZV3	Transferrin receptor protein 1 [OS=Sus scrofa]	0.902	9.18E-01
Q75NG9	Troponin T, fast skeletal muscle [OS=Sus scrofa]	0.901	9.18E-01
Q06AA4	U1 small nuclear ribonucleoprotein A [OS=Sus scrofa]	0.901	9.18E-01
A4Z6H0	Adenylosuccinate synthetase isozyme 1 [OS=Sus scrofa]	0.900	9.18E-01
Q9MZ16	Voltage-dependent anion-selective channel protein 1 [OS=Sus scrofa]	0.900	9.18E-01
Q06AB3	Ubiquitin carboxyl-terminal hydrolase isozyme L3 [OS=Sus scrofa]	0.899	9.18E-01
Q0PIT9	NAD(P)H-hydrate epimerase [OS=Sus scrofa]	0.897	9.18E-01
P50828	Hemopexin [OS=Sus scrofa]	0.895	9.18E-01
P12675	Calpastatin [OS=Sus scrofa]	0.893	9.18E-01
Q5PYH3	GTP-binding protein SAR1b [OS=Sus scrofa]	0.893	9.18E-01
A5GFY8	D-3-phosphoglycerate dehydrogenase [OS=Sus scrofa]	0.892	9.18E-01
P00355	Glyceraldehyde-3-phosphate dehydrogenase [OS=Sus scrofa]	0.891	9.18E-01
Q95274	Thymosin beta-4 [OS=Sus scrofa]	0.886	9.14E-01
Q2MJV9	Palmdelphin [OS=Sus scrofa]	0.885	9.14E-01
O77591	Inositol monophosphatase 1 [OS=Sus scrofa]	0.884	9.14E-01

P12026	Acyl-CoA-binding protein [OS=Sus scrofa]	0.883	9.14E-01
Q8SPJ9	Cytochrome c oxidase subunit 7A1, mitochondrial [OS=Sus scrofa]	0.875	8.96E-01
Q864V5	FUN14 domain-containing protein 2 [OS=Sus scrofa]	0.875	8.96E-01
Q2EN76	Nucleoside diphosphate kinase B [OS=Sus scrofa]	0.873	8.95E-01
Q04967	Heat shock 70 kDa protein 6 [OS=Sus scrofa]	0.869	8.92E-01
P81045	Cytochrome c oxidase copper chaperone [OS=Sus scrofa]	0.866	8.92E-01
P08835	Albumin [OS=Sus scrofa]	0.865	8.92E-01
Q29315	60S acidic ribosomal protein P2 [OS=Sus scrofa]	0.861	8.85E-01
O18934	Calsequestrin-2 [OS=Sus scrofa]	0.860	8.85E-01
P34935	Endoplasmic reticulum chaperone BiP [OS=Sus scrofa]	0.859	8.85E-01
P80272	Non-histone chromosomal protein HMG-17 [OS=Sus scrofa]	0.859	8.85E-01
Q5G6V9	Cofilin-2 [OS=Sus scrofa]	0.855	8.85E-01
Q29052	Inter-alpha-trypsin inhibitor heavy chain H1 [OS=Sus scrofa]	0.850	8.85E-01
A1XQR7	MICOS complex subunit MIC13 [OS=Sus scrofa]	0.841	8.83E-01
Q52NJ2	Ras-related protein Rab-1A [OS=Sus scrofa]	0.841	8.83E-01
P50578	Aldo-keto reductase family 1 member A1 [OS=Sus scrofa]	0.840	8.81E-01
P01846	Ig lambda chain C region [OS=Sus scrofa]	0.837	8.77E-01
P12309	Glutaredoxin-1 [OS=Sus scrofa]	0.834	8.77E-01
P06348	Histone H1t [OS=Sus scrofa]	0.833	8.77E-01
P80031	Glutathione S-transferase P [OS=Sus scrofa]	0.828	8.70E-01
P08132	Annexin A4 [OS=Sus scrofa]	0.822	8.54E-01
P00636	Fructose-1,6-bisphosphatase 1 [OS=Sus scrofa]	0.811	8.07E-01
Q7SIB7	Phosphoglycerate kinase 1 [OS=Sus scrofa]	0.803	7.88E-01
O97788	Fatty acid-binding protein, adipocyte [OS=Sus scrofa]	0.800	7.79E-01
Q08094	Calponin-2 [OS=Sus scrofa]	0.797	7.74E-01
P50390	Transthyretin [OS=Sus scrofa]	0.789	7.39E-01
A1XQU1	Proteasome subunit beta type-7 [OS=Sus scrofa]	0.784	7.29E-01
P79380	Metallothionein-2B [OS=Sus scrofa]	0.776	7.04E-01
Q5S1S4	Carbonic anhydrase 3 [OS=Sus scrofa]	0.765	6.83E-01
Q2HYU2	ATP-dependent 6-phosphofructokinase, muscle type [OS=Sus scrofa]	0.763	6.83E-01
P61958	Small ubiquitin-related modifier 2 [OS=Sus scrofa]	0.760	6.83E-01
Q29371	Triosephosphate isomerase [OS=Sus scrofa]	0.756	6.74E-01
Q1KYT0	Beta-enolase [OS=Sus scrofa]	0.754	6.69E-01

P08059	Glucose-6-phosphate isomerase [OS=Sus scrofa]	0.746	6.38E-01
P00571	Adenylate kinase isoenzyme 1 [OS=Sus scrofa]	0.741	6.15E-01
P16469	Polyunsaturated fatty acid lipoxygenase ALOX15 [OS=Sus scrofa]	0.736	5.96E-01
P81558	Myelin basic protein [OS=Sus scrofa]	0.733	5.87E-01
A5A8V7	Heat shock 70 kDa protein 1-like [OS=Sus scrofa]	0.699	4.61E-01
P80230	Enhancer of rudimentary homolog [OS=Sus scrofa]	0.696	4.56E-01
P00339	L-lactate dehydrogenase A chain [OS=Sus scrofa]	0.692	4.50E-01
Q3BDI7	Desmoglein-1 [OS=Sus scrofa]	0.681	4.13E-01
P43367	Calpain-2 catalytic subunit [OS=Sus scrofa]	0.637	2.53E-01
Q2EN75	Protein S100-A6 [OS=Sus scrofa]	0.634	2.45E-01
P27917	Apolipoprotein C-III [OS=Sus scrofa]	0.600	1.52E-01
P45846	Dermatopontin [OS=Sus scrofa]	0.594	1.40E-01
P02587	Troponin C, skeletal muscle [OS=Sus scrofa]	0.589	1.27E-01
Q8WNW3	Junction plakoglobin [OS=Sus scrofa]	0.535	5.23E-02

Table S2. The abundance ratio of adipose tissue proteins derived from suckling piglets.

UNIPROT Accession number	Description	Abundance Ratio: (soya) / (lino)	Abundance Ratio Adj. P-Value: (soya) / (lino)
Q95281	60S ribosomal protein L29 [OS=Sus scrofa]	100.00	1.18E-15
Q4PS85	Myozenin-1 [OS=Sus scrofa]	100.00	1.18E-15
F1RRT2	Myosin light chain 4 [OS=Sus scrofa]	6.93	2.47E-13
Q9TV62	Myosin-4 [OS=Sus scrofa]	5.12	3.33E-09
Q8SPS7	Haptoglobin [OS=Sus scrofa]	3.56	2.66E-05
P32195	Protegrin-2 [OS=Sus scrofa]	2.95	7.16E-04
Q29550	Liver carboxylesterase [OS=Sus scrofa]	0.51	1.10E-02
Q6RI85	Phosphoglycerate kinase 2 [OS=Sus scrofa]	0.52	1.49E-02
P06348	Histone H1t [OS=Sus scrofa]	2.37	2.20E-02
P51781	Glutathione S-transferase alpha M14 [OS=Sus scrofa]	2.34	2.48E-02
Q3BDI7	Desmoglein-1 [OS=Sus scrofa]	0.57	4.96E-02
Q29361	60S ribosomal protein L35 [OS=Sus scrofa]	2.15	6.26E-02
P79377	Metallothionein-1D [OS=Sus scrofa]	2.10	8.17E-02
O77696	Sarcoplasmic/endoplasmic reticulum calcium ATPase 3 [OS=Sus scrofa]	2.09	8.30E-02
P79263	Inter-alpha-trypsin inhibitor heavy chain H4 [OS=Sus scrofa]	2.08	8.57E-02

Q08092	Calponin-1 [OS=Sus scrofa]	0.66	0.2054
P79376	Metallothionein-1C [OS=Sus scrofa]	1.888	0.2169
P62863	40S ribosomal protein S30 [OS=Sus scrofa]	1.877	0.2214
Q9TSV6	V-type proton ATPase subunit G 2 [OS=Sus scrofa]	0.67	0.2214
Q29594	Creatine kinase B-type [OS=Sus scrofa]	0.675	0.2334
Q29197	40S ribosomal protein S9 [OS=Sus scrofa]	1.846	0.2587
Q29195	60S ribosomal protein L10 [OS=Sus scrofa]	1.836	0.2654
Q29293	60S ribosomal protein L3 [OS=Sus scrofa]	1.836	0.2654
Q5EE05	1-acylglycerol-3-phosphate O-acyltransferase ABHD5 [OS=Sus scrofa]	1.829	0.2680
P02067	Hemoglobin subunit beta [OS=Sus scrofa]	1.831	0.2680
Q75NG9	Troponin T, fast skeletal muscle [OS=Sus scrofa]	1.799	0.3084
Q5PXT2	LIM and cysteine-rich domains protein 1 [OS=Sus scrofa]	0.707	0.3321
P49923	Lipoprotein lipase [OS=Sus scrofa]	0.711	0.3462
Q9TV36	Fibrillin-1 [OS=Sus scrofa]	0.714	0.3577
Q29375	60S ribosomal protein L7a [OS=Sus scrofa]	1.75	0.3802
P01965	Hemoglobin subunit alpha [OS=Sus scrofa]	1.742	0.3860
Q6QRN9	ADP/ATP translocase 3 [OS=Sus scrofa]	1.734	0.3975
Q9TV61	Myosin-1 [OS=Sus scrofa]	1.727	0.4013
P53027	60S ribosomal protein L10a [OS=Sus scrofa]	1.716	0.4211
Q95332	Betaine--homocysteine S-methyltransferase 1 [OS=Sus scrofa]	1.697	0.4579
P79293	Myosin-7 [OS=Sus scrofa]	1.683	0.4931
Q95342	60S ribosomal protein L18 [OS=Sus scrofa]	1.674	0.5080
Q29529	Carbonyl reductase [NADPH] 2 [OS=Sus scrofa]	1.667	0.5244
P19133	Ferritin light chain [OS=Sus scrofa]	1.664	0.5288
P01025	Complement C3 [OS=Sus scrofa]	1.659	0.5390
Q29036	Dolichyl-diphosphooligosaccharide--protein glycosyltransferase subunit DAD1 [OS=Sus scrofa]	1.651	0.5534
Q29116	Tenascin [OS=Sus scrofa]	0.765	0.5603
P30034	Platelet factor 4 [OS=Sus scrofa]	1.64	0.5726
P15980	SLA class II histocompatibility antigen, DQ haplotype C alpha chain [OS=Sus scrofa]	1.629	0.5910
A1XQU3	60S ribosomal protein L14 [OS=Sus scrofa]	1.625	0.5934
I3LUP1	Mannose-1-phosphate guanyltransferase alpha [OS=Sus scrofa]	1.617	0.5972
F1RKQ4	Triokinase/FMN cyclase [OS=Sus scrofa]	1.615	0.5972
Q2EN81	ATP synthase subunit O, mitochondrial [OS=Sus scrofa]	1.61	0.6076
Q9TV63	Myosin-2 [OS=Sus scrofa]	0.788	0.6325

P81271	Myosin-11 [OS=Sus scrofa]	1.586	0.6534
O11780	Transforming growth factor-beta-induced protein ig-h3 [OS=Sus scrofa]	0.794	0.6534
Q9GKQ6	Biglycan [OS=Sus scrofa]	0.796	0.6660
P02587	Troponin C, skeletal muscle [OS=Sus scrofa]	1.576	0.6735
P18650	Apolipoprotein E [OS=Sus scrofa]	0.802	0.6747
Q6Q2C2	Bifunctional epoxide hydrolase 2 [OS=Sus scrofa]	0.8	0.6747
P05027	Sodium/potassium-transporting ATPase subunit beta-1 [OS=Sus scrofa]	0.801	0.6747
P49666	60S ribosomal protein L21 [OS=Sus scrofa]	1.566	0.6792
P51779	Complement factor D [OS=Sus scrofa]	0.813	0.7097
P11708	Malate dehydrogenase, cytoplasmic [OS=Sus scrofa]	0.813	0.7097
P45846	Dermatopontin [OS=Sus scrofa]	0.814	0.7108
Q2YGT9	60S ribosomal protein L6 [OS=Sus scrofa]	1.533	0.7212
P02540	Desmin [OS=Sus scrofa]	0.821	0.7212
Q29551	Succinyl-CoA:3-ketoacid coenzyme A transferase 1, mitochondrial [OS=Sus scrofa]	0.82	0.7212
Q3ZD69	Prelamin-A/C [OS=Sus scrofa]	0.823	0.7291
P20112	SPARC [OS=Sus scrofa]	0.827	0.7436
Q29558	NADP-dependent malic enzyme [OS=Sus scrofa]	0.83	0.7582
A1XQU5	60S ribosomal protein L27 [OS=Sus scrofa]	1.513	0.7614
P45845	Protein-lysine 6-oxidase [OS=Sus scrofa]	0.832	0.7614
P29804	Pyruvate dehydrogenase E1 component subunit alpha, somatic form, mitochondrial [OS=Sus scrofa]	0.832	0.7614
P62279	40S ribosomal protein S13 [OS=Sus scrofa]	1.507	0.7731
P27594	Interferon-induced GTP-binding protein Mx1 [OS=Sus scrofa]	1.506	0.7731
P16469	Polyunsaturated fatty acid lipoxygenase ALOX15 [OS=Sus scrofa]	1.506	0.7731
Q06AA3	Regucalcin [OS=Sus scrofa]	1.509	0.7731
P02554	Tubulin beta chain [OS=Sus scrofa]	0.838	0.7838
B1PK17	Aspartoacylase [OS=Sus scrofa]	0.841	0.7908
Q9XSD9	Decorin [OS=Sus scrofa]	0.845	0.8123
Q29052	Inter-alpha-trypsin inhibitor heavy chain H1 [OS=Sus scrofa]	1.484	0.8123
P81608	Salivary lipocalin [OS=Sus scrofa]	0.845	0.8123
A5A8V7	Heat shock 70 kDa protein 1-like [OS=Sus scrofa]	0.851	0.8126
Q71LE2	Histone H3.3 [OS=Sus scrofa]	1.482	0.8126
Q29384	Proteasome subunit beta type-4 [OS=Sus scrofa]	0.851	0.8126
Q1W674	Hexokinase-2 [OS=Sus scrofa]	1.468	0.8156

O02668	Inter-alpha-trypsin inhibitor heavy chain H2 [OS=Sus scrofa]	1.464	0.8156
P04163	Protein S100-A10 [OS=Sus scrofa]	0.86	0.8156
Q19AZ8	Prothrombin [OS=Sus scrofa]	1.466	0.8156
P02543	Vimentin [OS=Sus scrofa]	0.858	0.8156
Q8MJ14	Glutathione peroxidase 1 [OS=Sus scrofa]	0.862	0.8196
P12675	Calpastatin [OS=Sus scrofa]	0.865	0.8272
Q0Z8U2	40S ribosomal protein S3 [OS=Sus scrofa]	1.453	0.8311
P20305	Gelsolin [OS=Sus scrofa]	0.868	0.8311
A0A4X1UM84	Phosphoenolpyruvate carboxykinase, cytosolic [GTP] [OS=Sus scrofa]	1.451	0.8311
Q29290	Cystatin-B [OS=Sus scrofa]	0.873	0.8403
P29269	Myosin regulatory light polypeptide 9 [OS=Sus scrofa]	0.873	0.8403
D2WKD8	Sodium/potassium-transporting ATPase subunit alpha-2 [OS=Sus scrofa]	0.873	0.8403
P12309	Glutaredoxin-1 [OS=Sus scrofa]	0.875	0.8473
P18648	Apolipoprotein A-I [OS=Sus scrofa]	1.431	0.8507
P00172	Cytochrome b5 [OS=Sus scrofa]	0.881	0.8507
A1XQT2	Cytochrome c oxidase subunit 6C [OS=Sus scrofa]	1.432	0.8507
P33198	Isocitrate dehydrogenase [NADP], mitochondrial [OS=Sus scrofa]	1.432	0.8507
Q6QGC0	PDZ and LIM domain protein 3 [OS=Sus scrofa]	0.88	0.8507
Q29236	T-complex protein 1 subunit zeta [OS=Sus scrofa]	1.436	0.8507
Q29201	40S ribosomal protein S16 [OS=Sus scrofa]	1.424	0.8522
Q7M329	Ribonuclease T2 [OS=Sus scrofa]	1.423	0.8522
Q2VL90	Scavenger receptor cysteine-rich type 1 protein M130 [OS=Sus scrofa]	1.425	0.8522
P82460	Thioredoxin [OS=Sus scrofa]	0.883	0.8522
Q6SA96	40S ribosomal protein S23 [OS=Sus scrofa]	1.41	0.8528
Q29214	60S acidic ribosomal protein P0 [OS=Sus scrofa]	1.422	0.8528
Q6QAT0	60S ribosomal protein L32 [OS=Sus scrofa]	1.395	0.8528
Q95276	60S ribosomal protein L5 [OS=Sus scrofa]	1.412	0.8528
P36887	cAMP-dependent protein kinase catalytic subunit alpha [OS=Sus scrofa]	0.895	0.8528
O62839	Catalase [OS=Sus scrofa]	0.898	0.8528
P00795	Cathepsin D [OS=Sus scrofa]	0.9	0.8528
O62680	CD59 glycoprotein [OS=Sus scrofa]	0.895	0.8528
P60982	Destrin [OS=Sus scrofa]	0.89	0.8528
P79381	Epoxide hydrolase 1 [OS=Sus scrofa]	1.42	0.8528
P49924	Fatty acid-binding protein, liver [OS=Sus scrofa]	1.4	0.8528
P46410	Glutamine synthetase [OS=Sus scrofa]	1.419	0.8528

Q863Z0	Proteasome activator complex subunit 2 [OS=Sus scrofa]	1.416	0.8528
P80895	Protein-L-isoaspartate(D-aspartate) O-methyltransferase [OS=Sus scrofa]	0.895	0.8528
O46560	Pyridoxal kinase [OS=Sus scrofa]	1.402	0.8528
A2TLM1	Ribose-5-phosphate isomerase [OS=Sus scrofa]	0.893	0.8528
Q6DUB7	Stathmin [OS=Sus scrofa]	1.409	0.8528
Q29371	Triosephosphate isomerase [OS=Sus scrofa]	0.894	0.8528
Q9N2D1	Tryptase [OS=Sus scrofa]	0.893	0.8528
Q06AA9	Ubiquitin-conjugating enzyme E2 D2 [OS=Sus scrofa]	1.396	0.8528
P19620	Annexin A2 [OS=Sus scrofa]	0.906	0.8540
P80229	Leukocyte elastase inhibitor [OS=Sus scrofa]	1.392	0.8540
P50578	Aldo-keto reductase family 1 member A1 [OS=Sus scrofa]	0.91	0.8665
Q29041	Ficolin-2 [OS=Sus scrofa]	1.377	0.8665
Q2PKF4	Guanine nucleotide-binding protein G(q) subunit alpha [OS=Sus scrofa]	0.914	0.8665
P50828	Hemopexin [OS=Sus scrofa]	1.383	0.8665
Q06A98	Serine/arginine-rich splicing factor 2 [OS=Sus scrofa]	0.912	0.8665
P15983	SLA class II histocompatibility antigen, DQ haplotype D beta chain [OS=Sus scrofa]	1.378	0.8665
P48819	Vitronectin [OS=Sus scrofa]	1.378	0.8665
P27917	Apolipoprotein C-III [OS=Sus scrofa]	1.375	0.8666
Q29549	Clusterin [OS=Sus scrofa]	1.376	0.8666
Q8WNV7	Dehydrogenase/reductase SDR family member 4 [OS=Sus scrofa]	1.376	0.8666
P80230	Enhancer of rudimentary homolog [OS=Sus scrofa]	1.375	0.8668
Q6S4N2	Heat shock 70 kDa protein 1B [OS=Sus scrofa]	0.919	0.8771
Q7YS99	Optineurin [OS=Sus scrofa]	0.92	0.8771
P09571	Serotransferrin [OS=Sus scrofa]	0.919	0.8771
Q95274	Thymosin beta-4 [OS=Sus scrofa]	1.366	0.8801
P28839	Cytosol aminopeptidase [OS=Sus scrofa]	1.354	0.8866
Q9GL01	Dolichyl-diphosphooligosaccharide--protein glycosyltransferase subunit 2 [OS=Sus scrofa]	1.357	0.8866
Q9TUI8	Fatty-acid amide hydrolase 1 [OS=Sus scrofa]	1.359	0.8866
Q9GLP0	Integrin beta-1 [OS=Sus scrofa]	0.926	0.8866
P36968	Phospholipid hydroperoxide glutathione peroxidase [OS=Sus scrofa]	0.929	0.8866
P04366	Protein AMBP [OS=Sus scrofa]	1.363	0.8866
P10775	Ribonuclease inhibitor [OS=Sus scrofa]	0.928	0.8866

Q29024	Spliceosome RNA helicase DDX39B [OS=Sus scrofa]	1.356	0.8866
P62272	40S ribosomal protein S18 [OS=Sus scrofa]	1.336	0.9018
P49171	40S ribosomal protein S26 [OS=Sus scrofa]	1.331	0.9018
P08835	Albumin [OS=Sus scrofa]	0.937	0.9018
Q7M2W6	Alpha-crystallin B chain [OS=Sus scrofa]	1.337	0.9018
P08132	Annexin A4 [OS=Sus scrofa]	0.944	0.9018
Q95ND5	Caspase-3 [OS=Sus scrofa]	1.33	0.9018
A1E295	Cathepsin B [OS=Sus scrofa]	0.936	0.9018
Q5G6V9	Cofilin-2 [OS=Sus scrofa]	0.94	0.9018
Q29381	Dolichyl-diphosphooligosaccharide--protein glycosyltransferase 48 kDa subunit [OS=Sus scrofa]	1.349	0.9018
A6M931	Eukaryotic initiation factor 4A-III [OS=Sus scrofa]	1.331	0.9018
O97788	Fatty acid-binding protein, adipocyte [OS=Sus scrofa]	0.94	0.9018
O02772	Fatty acid-binding protein, heart [OS=Sus scrofa]	0.938	0.9018
P12682	High mobility group protein B1 [OS=Sus scrofa]	1.335	0.9018
P62802	Histone H4 [OS=Sus scrofa]	1.339	0.9018
P81693	Low molecular weight phosphotyrosine protein phosphatase [OS=Sus scrofa]	0.942	0.9018
Q8MJ39	Matrix Gla protein [OS=Sus scrofa]	1.336	0.9018
Q9TSX9	Peroxiredoxin-6 [OS=Sus scrofa]	0.942	0.9018
Q64L94	Proteasome activator complex subunit 1 [OS=Sus scrofa]	1.332	0.9018
A1XQU1	Proteasome subunit beta type-7 [OS=Sus scrofa]	0.946	0.9018
Q29277	Protein phosphatase 1 regulatory subunit 1B [OS=Sus scrofa]	0.938	0.9018
B3SP85	Gamma-interferon-inducible-lysosomal thiol reductase [OS=Sus scrofa]	0.949	0.9113
P00355	Glyceraldehyde-3-phosphate dehydrogenase [OS=Sus scrofa]	0.951	0.9170
Q2QLE2	Caveolin-2 [OS=Sus scrofa]	0.952	0.9237
Q07717	Beta-2-microglobulin [OS=Sus scrofa]	1.32	0.9266
Q29243	Dystroglycan [OS=Sus scrofa]	0.955	0.9266
Q6J1I8	E3 ubiquitin-protein ligase RNF114 [OS=Sus scrofa]	0.954	0.9266
P15145	Aminopeptidase N [OS=Sus scrofa]	1.317	0.9296
P62901	60S ribosomal protein L31 [OS=Sus scrofa]	1.31	0.9390
P80031	Glutathione S-transferase P [OS=Sus scrofa]	0.961	0.9390
Q45FY6	Hypoxanthine-guanine phosphoribosyltransferase [OS=Sus scrofa]	1.313	0.9390

P79380	Metallothionein-2B [OS=Sus scrofa]	0.962	0.9390
P31950	Protein S100-A11 [OS=Sus scrofa]	1.311	0.9390
P80276	Aldo-keto reductase family 1 member B1 [OS=Sus scrofa]	0.963	0.9396
P11607	Sarcoplasmic/endoplasmic reticulum calcium ATPase 2 [OS=Sus scrofa]	1.308	0.9396
P63221	40S ribosomal protein S21 [OS=Sus scrofa]	0.965	0.9418
P79324	60S ribosomal protein L15 [OS=Sus scrofa]	1.29	0.9418
P67985	60S ribosomal protein L22 [OS=Sus scrofa]	1.291	0.9418
P50447	Alpha-1-antitrypsin [OS=Sus scrofa]	1.29	0.9418
P29700	Alpha-2-HS-glycoprotein [OS=Sus scrofa]	1.298	0.9418
P37111	Aminoacylase-1 [OS=Sus scrofa]	0.973	0.9418
P28491	Calreticulin [OS=Sus scrofa]	0.977	0.9418
Q5S3G4	Cytochrome c oxidase subunit 5B, mitochondrial [OS=Sus scrofa]	0.975	0.9418
A0PFK7	F-actin-capping protein subunit beta [OS=Sus scrofa]	0.97	0.9418
P00636	Fructose-1,6-bisphosphatase 1 [OS=Sus scrofa]	1.305	0.9418
Q9N1F5	Glutathione S-transferase omega-1 [OS=Sus scrofa]	1.289	0.9418
P01846	Ig lambda chain C region [OS=Sus scrofa]	0.969	0.9418
P14632	Lactotransferrin [OS=Sus scrofa]	1.296	0.9418
P17560	N-acylglucosamine 2-epimerase [OS=Sus scrofa]	0.972	0.9418
Q29099	Polypyrimidine tract-binding protein 1 [OS=Sus scrofa]	1.291	0.9418
Q52NJ6	Ras-related protein Rab-14 [OS=Sus scrofa]	0.975	0.9418
M3TYT0	Rho-associated protein kinase 2 [OS=Sus scrofa]	1.294	0.9418
Q29090	Serine/threonine-protein phosphatase 2A 55 kDa regulatory subunit B alpha isoform [OS=Sus scrofa]	0.966	0.9418
Q9GJT2	S-formylglutathione hydrolase [OS=Sus scrofa]	0.968	0.9418
Q764M5	Signal transducer and activator of transcription 1 [OS=Sus scrofa]	1.289	0.9418
P53590	Succinate--CoA ligase [GDP-forming] subunit beta, mitochondrial [OS=Sus scrofa]	0.971	0.9418
Q29554	Trifunctional enzyme subunit alpha, mitochondrial [OS=Sus scrofa]	1.3	0.9418
Q2XQV4	Aldehyde dehydrogenase, mitochondrial [OS=Sus scrofa]	0.978	0.9449
Q03472	Apolipoprotein R [OS=Sus scrofa]	0.98	0.9449
Q9TTB4	Fibromodulin [OS=Sus scrofa]	1.281	0.9482
P80310	Protein S100-A12 [OS=Sus scrofa]	1.282	0.9482
Q06AB3	Ubiquitin carboxyl-terminal hydrolase isozyme L3 [OS=Sus scrofa]	0.982	0.9482

Q29561	UMP-CMP kinase [OS=Sus scrofa]	0.981	0.9482
P59083	14 kDa phosphohistidine phosphatase [OS=Sus scrofa]	0.987	0.9503
Q95339	ATP synthase subunit f, mitochondrial [OS=Sus scrofa]	1.278	0.9503
Q9GMB0	Dolichyl-diphosphooligosaccharide--protein glycosyltransferase subunit 1 [OS=Sus scrofa]	1.279	0.9503
P10173	Fumarate hydratase, mitochondrial [OS=Sus scrofa]	1.27	0.9503
P29797	Guanine nucleotide-binding protein G(s) subunit alpha [OS=Sus scrofa]	1.272	0.9503
P79382	Microsomal glutathione S-transferase 1 [OS=Sus scrofa]	0.99	0.9503
Q0PIT9	NAD(P)H-hydrate epimerase [OS=Sus scrofa]	0.986	0.9503
P23220	Plasma membrane calcium-transporting ATPase 1 [OS=Sus scrofa]	1.278	0.9503
P54612	Serine/threonine-protein phosphatase 2A 65 kDa regulatory subunit A alpha isoform [OS=Sus scrofa]	1.27	0.9503
Q007T0	Succinate dehydrogenase [ubiquinone] iron-sulfur subunit, mitochondrial [OS=Sus scrofa]	0.986	0.9503
P26234	Vinculin [OS=Sus scrofa]	0.989	0.9503
Q007T2	Cell division control protein 42 homolog [OS=Sus scrofa]	1.266	0.9626
P16276	Aconitate hydratase, mitochondrial [OS=Sus scrofa]	0.996	0.9645
Q6Q2J0	Amine oxidase [flavin-containing] A [OS=Sus scrofa]	1.256	0.9645
P00506	Aspartate aminotransferase, mitochondrial [OS=Sus scrofa]	1.258	0.9645
P04574	Calpain small subunit 1 [OS=Sus scrofa]	0.999	0.9645
P61220	Eukaryotic translation initiation factor 1b [OS=Sus scrofa]	1.002	0.9645
P31006	Guanylate kinase [OS=Sus scrofa]	0.999	0.9645
Q5ISC6	Heme-binding protein 1 [OS=Sus scrofa]	0.998	0.9645
P62936	Peptidyl-prolyl cis-trans isomerase A [OS=Sus scrofa]	0.996	0.9645
P26044	Radixin [OS=Sus scrofa]	1.262	0.9645
Q3YLA6	Serine/arginine-rich splicing factor 1 [OS=Sus scrofa]	1.002	0.9645
Q9TV69	Trans-1,2-dihydrobenzene-1,2-diol dehydrogenase [OS=Sus scrofa]	1.258	0.9645
Q9MZ15	Voltage-dependent anion-selective channel protein 2 [OS=Sus scrofa]	1.257	0.9645

P15468	Ribonuclease 4 [OS=Sus scrofa]	1.003	0.9671
Q863I2	Serine/threonine-protein kinase OSR1 [OS=Sus scrofa]	1.004	0.9671
P05024	Sodium/potassium-transporting ATPase subunit alpha-1 [OS=Sus scrofa]	1.252	0.9741
Q767L7	Tubulin beta chain [OS=Sus scrofa]	1.006	0.9743
Q6QAP7	40S ribosomal protein S17 [OS=Sus scrofa]	1.009	0.9775
Q29315	60S acidic ribosomal protein P2 [OS=Sus scrofa]	1.009	0.9775
P55931	Electron transfer flavoprotein-ubiquinone oxidoreductase, mitochondrial [OS=Sus scrofa]	1.008	0.9775
Q95250	Membrane-associated progesterone receptor component 1 [OS=Sus scrofa]	1.008	0.9775
P23687	Prolyl endopeptidase [OS=Sus scrofa]	1.008	0.9775
P06867	Plasminogen [OS=Sus scrofa]	1.247	0.9780
A1XQU9	40S ribosomal protein S20 [OS=Sus scrofa]	1.24	0.9791
P00571	Adenylate kinase isoenzyme 1 [OS=Sus scrofa]	1.032	0.9791
P39036	Alpha-S2-casein [OS=Sus scrofa]	1.223	0.9791
P19619	Annexin A1 [OS=Sus scrofa]	1.031	0.9791
P80021	ATP synthase subunit alpha, mitochondrial [OS=Sus scrofa]	1.237	0.9791
Q9MYT8	ATP synthase subunit e, mitochondrial [OS=Sus scrofa]	1.029	0.9791
Q29307	ATPase inhibitor, mitochondrial [OS=Sus scrofa]	1.032	0.9791
D2SW95	Coatomer subunit beta [OS=Sus scrofa]	1.245	0.9791
P10668	Cofilin-1 [OS=Sus scrofa]	1.026	0.9791
Q03710	Complement factor B [OS=Sus scrofa]	1.24	0.9791
Q5XLD3	Creatine kinase M-type [OS=Sus scrofa]	1.226	0.9791
A5GFY8	D-3-phosphoglycerate dehydrogenase [OS=Sus scrofa]	1.029	0.9791
P34935	Endoplasmic reticulum chaperone BiP [OS=Sus scrofa]	1.013	0.9791
P34930	Heat shock 70 kDa protein 1A [OS=Sus scrofa]	1.029	0.9791
Q04967	Heat shock 70 kDa protein 6 [OS=Sus scrofa]	1.235	0.9791
P0C5I2	Mannose-1-phosphate guanyltransferase beta [OS=Sus scrofa]	1.239	0.9791
Q866Y3	N-acetylmuramoyl-L-alanine amidase [OS=Sus scrofa]	1.24	0.9791
Q2EN76	Nucleoside diphosphate kinase B [OS=Sus scrofa]	1.239	0.9791
Q9GL51	Platelet-activating factor acetylhydrolase IB subunit alpha [OS=Sus scrofa]	1.227	0.9791
P0DTA4	Propionyl-CoA carboxylase alpha chain, mitochondrial [OS=Sus scrofa]	1.026	0.9791
Q52NJ1	Ras-related protein Rab-11A [OS=Sus scrofa]	1.225	0.9791

Q0QF01	Succinate dehydrogenase [ubiquinone] flavoprotein subunit, mitochondrial [OS=Sus scrofa]	1.014	0.9791
O97580	Succinate--CoA ligase [ADP-forming] subunit beta, mitochondrial [OS=Sus scrofa]	1.229	0.9791
P47788	Thimet oligopeptidase [OS=Sus scrofa]	1.226	0.9791
Q2XVP4	Tubulin alpha-1B chain [OS=Sus scrofa]	1.024	0.9791
Q29380	Voltage-dependent anion-selective channel protein 3 [OS=Sus scrofa]	1.221	0.9791
Q06AU6	Ras-related protein Rab-5A [OS=Sus scrofa]	1.033	0.9794
Q29599	2'-5'-oligoadenylate synthase 1 [OS=Sus scrofa]	1.213	0.9801
Q28960	Carbonyl reductase [NADPH] 1 [OS=Sus scrofa]	1.209	0.9801
P19130	Ferritin heavy chain [OS=Sus scrofa]	1.034	0.9801
P42174	Glutamate dehydrogenase 1, mitochondrial [OS=Sus scrofa]	1.036	0.9801
P17741	High mobility group protein B2 [OS=Sus scrofa]	1.213	0.9801
P60662	Myosin light polypeptide 6 [OS=Sus scrofa]	1.037	0.9801
P63246	Receptor of activated protein C kinase 1 [OS=Sus scrofa]	1.213	0.9801
P27485	Retinol-binding protein 4 [OS=Sus scrofa]	1.042	0.9801
P03974	Transitional endoplasmic reticulum ATPase [OS=Sus scrofa]	1.034	0.9801
A5GFS8	Vesicle-associated membrane protein-associated protein B [OS=Sus scrofa]	1.211	0.9801
Q9GLP2	Vitamin K-dependent protein C [OS=Sus scrofa]	1.039	0.9801
Q8MJ49	Osteoclast-stimulating factor 1 [OS=Sus scrofa]	1.044	0.9838
Q29228	4-trimethylaminobutyraldehyde dehydrogenase [OS=Sus scrofa]	1.051	0.9858
P14332	6-phosphogluconate dehydrogenase, decarboxylating [OS=Sus scrofa]	1.06	0.9858
P19205	Acylamino-acid-releasing enzyme [OS=Sus scrofa]	1.058	0.9858
P12026	Acyl-CoA-binding protein [OS=Sus scrofa]	1.194	0.9858
Q99028	Catechol O-methyltransferase [OS=Sus scrofa]	1.061	0.9858
O97507	Coagulation factor XII [OS=Sus scrofa]	1.203	0.9858
Q69DK8	Complement C1s subcomponent [OS=Sus scrofa]	1.06	0.9858
A7Y521	COP9 signalosome complex subunit 4 [OS=Sus scrofa]	1.055	0.9858
A7TX81	COP9 signalosome complex subunit 6 [OS=Sus scrofa]	1.201	0.9858
P09623	Dihydrolipoyl dehydrogenase, mitochondrial [OS=Sus scrofa]	1.065	0.9858
Q8MJ30	Dihydropteridine reductase [OS=Sus scrofa]	1.054	0.9858
Q5GN48	Dystrophin [OS=Sus scrofa]	1.065	0.9858
Q29387	Elongation factor 1-gamma [OS=Sus scrofa]	1.196	0.9858

Q9GKX6	Galactose mutarotase [OS=Sus scrofa]	1.059	0.9858
Q49I35	Galectin-1 [OS=Sus scrofa]	1.051	0.9858
P81140	Glutaryl-CoA dehydrogenase, mitochondrial [OS=Sus scrofa]	1.19	0.9858
O02705	Heat shock protein HSP 90-alpha [OS=Sus scrofa]	1.202	0.9858
O77591	Inositol monophosphatase 1 [OS=Sus scrofa]	1.195	0.9858
Q8WNW3	Junction plakoglobin [OS=Sus scrofa]	1.185	0.9858
P00336	L-lactate dehydrogenase B chain [OS=Sus scrofa]	1.055	0.9858
Q5BLZ2	Perilipin-3 [OS=Sus scrofa]	1.187	0.9858
Q7SIB7	Phosphoglycerate kinase 1 [OS=Sus scrofa]	1.052	0.9858
Q56P28	PRA1 family protein 3 [OS=Sus scrofa]	1.2	0.9858
Q8MIK9	Protein phosphatase 1 regulatory subunit 14B [OS=Sus scrofa]	1.051	0.9858
Q5W9D5	Protein quaking [OS=Sus scrofa]	1.055	0.9858
Q52NJ2	Ras-related protein Rab-1A [OS=Sus scrofa]	1.202	0.9858
Q06AU7	Ras-related protein Rab-1B [OS=Sus scrofa]	1.189	0.9858
P61958	Small ubiquitin-related modifier 2 [OS=Sus scrofa]	1.046	0.9858
P61288	Translationally-controlled tumor protein [OS=Sus scrofa]	1.063	0.9858
P67937	Tropomyosin alpha-4 chain [OS=Sus scrofa]	1.183	0.9858
Q28833	von Willebrand factor [OS=Sus scrofa]	1.06	0.9858
Q29048	V-type proton ATPase catalytic subunit A [OS=Sus scrofa]	1.184	0.9858
Q9MZ16	Voltage-dependent anion-selective channel protein 1 [OS=Sus scrofa]	1.066	0.9864
P05207	cAMP-dependent protein kinase type II-alpha regulatory subunit [OS=Sus scrofa]	1.069	0.9936
P62197	26S proteasome regulatory subunit 8 [OS=Sus scrofa]	1.161	0.9957
P46405	40S ribosomal protein S12 [OS=Sus scrofa]	1.168	0.9957
Q4GWZ2	40S ribosomal protein SA [OS=Sus scrofa]	1.077	0.9957
Q29205	60S ribosomal protein L11 [OS=Sus scrofa]	1.083	0.9957
Q95307	60S ribosomal protein L13a [OS=Sus scrofa]	1.075	0.9957
Q710C4	Adenosylhomocysteinase [OS=Sus scrofa]	1.166	0.9957
Q52NJ4	ADP-ribosylation factor-like protein 3 [OS=Sus scrofa]	1.158	0.9957
P00889	Citrate synthase, mitochondrial [OS=Sus scrofa]	1.074	0.9957
P50667	Cytochrome c oxidase subunit 2 [OS=Sus scrofa]	1.088	0.9957
Q9N0F1	Dihydrolipoyllysine-residue succinyltransferase component of 2-oxoglutarate dehydrogenase complex, mitochondrial [OS=Sus scrofa]	1.175	0.9957
Q29092	Endoplasmin [OS=Sus scrofa]	1.173	0.9957

P08059	Glucose-6-phosphate isomerase [OS=Sus scrofa]	1.158	0.9957
Q5PYH3	GTP-binding protein SAR1b [OS=Sus scrofa]	1.084	0.9957
Q68J42	Hormone-sensitive lipase [OS=Sus scrofa]	1.173	0.9957
Q29545	Inhibitor of carbonic anhydrase [OS=Sus scrofa]	1.16	0.9957
Q8SQ26	Lambda-crystallin homolog [OS=Sus scrofa]	1.077	0.9957
P00346	Malate dehydrogenase, mitochondrial [OS=Sus scrofa]	1.087	0.9957
P52552	Peroxiredoxin-2 [OS=Sus scrofa]	1.073	0.9957
P79281	Pleiotrophin [OS=Sus scrofa]	1.081	0.9957
Q19PY3	RNA-splicing ligase RtcB homolog [OS=Sus scrofa]	1.071	0.9957
P67776	Serine/threonine-protein phosphatase 2A catalytic subunit alpha isoform [OS=Sus scrofa]	1.085	0.9957
P61292	Serine/threonine-protein phosphatase PP1-beta catalytic subunit [OS=Sus scrofa]	1.077	0.9957
P15982	SLA class II histocompatibility antigen, DQ haplotype C beta chain [OS=Sus scrofa]	1.159	0.9957
P04178	Superoxide dismutase [Cu-Zn] [OS=Sus scrofa]	1.073	0.9957
P28768	Superoxide dismutase [Mn], mitochondrial [OS=Sus scrofa]	1.07	0.9957
Q6SEG5	Ubiquitin carboxyl-terminal hydrolase isozyme L1 [OS=Sus scrofa]	1.159	0.9957
P79303	UTP--glucose-1-phosphate uridylyltransferase [OS=Sus scrofa]	1.072	0.9957
Q29221	F-actin-capping protein subunit alpha-2 [OS=Sus scrofa]	1.088	0.9961
P05383	cAMP-dependent protein kinase catalytic subunit beta [OS=Sus scrofa]	1.156	0.9967
P68137	Actin, alpha skeletal muscle [OS=Sus scrofa]	1.155	0.9970
O46409	Apolipoprotein A-IV [OS=Sus scrofa]	1.154	0.9970
Q29350	Splicing factor U2AF 35 kDa subunit [OS=Sus scrofa]	1.091	0.9970
P07802	cAMP-dependent protein kinase type I-alpha regulatory subunit [OS=Sus scrofa]	1.152	0.9976
Q6QAT1	40S ribosomal protein S28 [OS=Sus scrofa]	1.106	0.9978
P00503	Aspartate aminotransferase, cytoplasmic [OS=Sus scrofa]	1.1	0.9978
O02840	Cadherin-5 [OS=Sus scrofa]	1.098	0.9978
P35750	Calpain-1 catalytic subunit [OS=Sus scrofa]	1.144	0.9978
P43367	Calpain-2 catalytic subunit [OS=Sus scrofa]	1.094	0.9978
Q5S1S4	Carbonic anhydrase 3 [OS=Sus scrofa]	1.096	0.9978
Q6RVA9	Caveolin-1 [OS=Sus scrofa]	1.101	0.9978

Q6UAQ8	Electron transfer flavoprotein subunit beta [OS=Sus scrofa]	1.107	0.9978
B6CVD7	ERO1-like protein alpha [OS=Sus scrofa]	1.104	0.9978
P14460	Fibrinogen alpha chain [OS=Sus scrofa]	1.148	0.9978
P00348	Hydroxyacyl-coenzyme A dehydrogenase, mitochondrial [OS=Sus scrofa]	1.098	0.9978
P00339	L-lactate dehydrogenase A chain [OS=Sus scrofa]	1.105	0.9978
P80928	Macrophage migration inhibitory factor [OS=Sus scrofa]	1.095	0.9978
P41367	Medium-chain specific acyl-CoA dehydrogenase, mitochondrial [OS=Sus scrofa]	1.096	0.9978
P26042	Moesin [OS=Sus scrofa]	1.142	0.9978
P04175	NADPH--cytochrome P450 reductase [OS=Sus scrofa]	1.102	0.9978
Q2MJV8	Paralemmin-1 [OS=Sus scrofa]	1.138	0.9978
Q95242	Platelet endothelial cell adhesion molecule [OS=Sus scrofa]	1.144	0.9978
P79384	Propionyl-CoA carboxylase beta chain, mitochondrial [OS=Sus scrofa]	1.098	0.9978
Q6Q7J2	Rab GDP dissociation inhibitor beta [OS=Sus scrofa]	1.107	0.9978
P79273	Short-chain specific acyl-CoA dehydrogenase, mitochondrial [OS=Sus scrofa]	1.094	0.9978
O19069	Succinate--CoA ligase [ADP/GDP-forming] subunit alpha, mitochondrial [OS=Sus scrofa]	1.139	0.9978
P42639	Tropomyosin alpha-1 chain [OS=Sus scrofa]	1.142	0.9978
P02550	Tubulin alpha-1A chain [OS=Sus scrofa]	1.152	0.9978
Q8MIZ3	m7GpppX diphosphatase [OS=Sus scrofa]	1.137	0.9983
Q9XT90	Matrix metalloproteinase-14 [OS=Sus scrofa]	1.136	0.9990
Q29308	40S ribosomal protein S19 [OS=Sus scrofa]	1.125	0.9993
P62831	60S ribosomal protein L23 [OS=Sus scrofa]	1.116	0.9993
Q767L0	ATP-binding cassette sub-family F member 1 [OS=Sus scrofa]	1.115	0.9993
Q1KYT0	Beta-enolase [OS=Sus scrofa]	1.122	0.9993
Q29238	Chloride intracellular channel protein 1 [OS=Sus scrofa]	1.122	0.9993
Q95283	Cytochrome c oxidase subunit 4 isoform 1, mitochondrial [OS=Sus scrofa]	1.118	0.9993
Q29042	Ficolin-1 [OS=Sus scrofa]	1.133	0.9993
Q2MJK3	GTPase NRas [OS=Sus scrofa]	1.129	0.9993
Q52NJ3	GTP-binding protein SAR1a [OS=Sus scrofa]	1.115	0.9993
Q5S1U1	Heat shock protein beta-1 [OS=Sus scrofa]	1.12	0.9993

B0KYV5	LIM domain and actin-binding protein 1 [OS=Sus scrofa]	1.114	0.9993
P79274	Long-chain specific acyl-CoA dehydrogenase, mitochondrial [OS=Sus scrofa]	1.132	0.9993
P83686	NADH-cytochrome b5 reductase 3 [OS=Sus scrofa]	1.127	0.9993
P79403	Neutral alpha-glucosidase AB [OS=Sus scrofa]	1.124	0.9993
F1RQM2	Phosphoacetylglucosamine mutase [OS=Sus scrofa]	1.122	0.9993
Q29073	Prostaglandin reductase 1 [OS=Sus scrofa]	1.113	0.9993
A5GFQ5	Protein canopy homolog 3 [OS=Sus scrofa]	1.112	0.9993
O02858	Stearoyl-CoA desaturase [OS=Sus scrofa]	1.111	0.9993
P50390	Transthyretin [OS=Sus scrofa]	1.12	0.9993
A1XQV4	Tropomyosin alpha-3 chain [OS=Sus scrofa]	1.13	0.9993
